# Results of Late Gadolinium Enhancement in Children Affected by Dilated Cardiomyopathy

**DOI:** 10.3389/fped.2017.00013

**Published:** 2017-02-06

**Authors:** Giuseppe Muscogiuri, Paolo Ciliberti, Domenico Mastrodicasa, Marcello Chinali, Gabriele Rinelli, Teresa Pia Santangelo, Carmela Napolitano, Benedetta Leonardi, Aurelio Secinaro

**Affiliations:** ^1^Department of Imaging, Bambino Gesù – Children’s Hospital IRCCS, Rome, Italy; ^2^Department of Clinical and Molecular Medicine, University of Rome “Sapienza”, Rome, Italy; ^3^Department of Pediatric Cardiology and Cardiac Surgery, Bambino Gesù – Children’s Hospital IRCCS, Rome, Italy; ^4^Department of Neurosciences, Imaging and Clinical Sciences, Diagnostic Imaging and Therapy, University “G. D’Annunzio”, Chieti, Italy

**Keywords:** dilated cardiomyopathy, late gadolinium enhancement, cardiac magnetic resonance, systolic function, ventricular mechanics

## Abstract

**Background:**

Little is known about the clinical value of late gadolinium enhancement (LGE), in children affected by dilated cardiomyopathy (DCM).

**Materials and methods:**

We retrospectively evaluated 15 patients (8 ± 6 years, 6 males) with diagnosis of DCM who underwent cardiac magnetic resonance since 2014. All scans were performed with a 1.5 T system (Aera, Siemens). Study protocol included cine steady-state free precession sequences, followed by administration of 0.2 mmol/kg of gadolinium-based contrast agent. Inversion recovery Turbo Flash sequences, in the same position of cine images, were acquired 10–15 min after the injection of contrast agent, in order to assess the presence of LGE. The latter was considered positive with a signal intensity >6 SD from normal myocardial tissue. Indexed end-diastolic volume (EDVi) and end-systolic volume (ESVi), and left ventricle (LV) ejection fraction (EF) were calculated by using dedicated software on off-line workstation. Global longitudinal strain and diastolic function were evaluated by echocardiography. Clinical follow-up, including death, transplant, and listing for heart transplant [major adverse cardiac events (MACE)], were evaluated. Patients were divided into two different subgroups: negative (Group A) and positive (Group B) for presence of LGE. Statistical analysis was performed by using Mann–Whitney *U* test (*p* < 0.05 considered as statistically significant).

**Results:**

Seven patients (47%) showed LGE. A global diffuse subendocardial pattern was evident in all patients presenting LGE (7/7, 100%). The following main LV indexes were observed in the two subgroups. Group A: EDVi = 96 ± 33 ml, ESVi = 56 ± 29 ml, LV EF = 45 ± 10%, global longitudinal strain = −16 ± 5%, *E/e*′ ratio = 10 ± 3, MACE = 1. Group B: EDVi = 130 ± 60 ml, ESVi = 89 ± 43 ml, LV EF = 31 ± 6%, global longitudinal strain = −13 ± 4%, *E/e*′ ratio = 9 ± 3, MACE = 3. There was no statistically significant difference between the two groups, in terms of EDVi (*p*: 0.2), ESVi (*p*: 0.2), and *E/e*′ ratio (0.9), whereas a significant difference of LV EF, presence of significative mitral regurgitation, and global longitudinal strain were observed (respectively, *p*: 0.03, *p*: 0.009, and *p*: 0.03).

**Conclusion:**

In our population of children with DCM, LGE shows a global diffuse subendocardial pattern. Presence of LGE seems to play a role in these patients determining a worst global systolic function.

## Introduction

Dilated cardiomyopathy (DCM) is defined as a ventricular dilatation associated with systolic dysfunction not secondary to other cardiac abnormalities such as coronary artery disease, valvular, or congenital heart disease. Incidence of DCM in children is extremely rare (0:58 cases/100,000 children) (1).

Etiology of dilation is known in about three thirds of cases; however, the majority remains without a confirmed diagnosis (1, 2).

Dilated cardiomyopathy development in pediatric patients may be either genetic linked, if genetic pedigree of the family is responsible for it, or non-familiar (1). DCM in children could be expression of neuromuscular disorders, typically characterized by dystrophin mutations. Finally, DCM can be linked to congenital errors of metabolism such as lysosomal storage diseases, carnitine deficiency, and mitochondrial myopathies (3).

Ventricular dilatation may also be due to “non-familiar” conditions. In these cases, DCM is usually the consequence of a previous myocarditis, chemotherapy, or fulminant Kawasaki (1).

Clinical manifestations of DCM can range from asymptomatic patients up to heart failure and development of malignant arrhythmias (4). If medical treatment fails, patients are listed for cardiac transplantation, or must receive mechanical circulatory support.

Cardiac magnetic resonance (CMR) has gained a crucial role in adults with DCM, since CMR provides reliable information about cardiac function and muscle tissue characterization (5). In fact, CMR is able to evaluate with great accuracy regional left ventricle (LV) wall motion, global systolic function, and presence of myocardial fibrosis (6, 7). Late gadolinium enhancement (LGE) imaging enables identification and quantification of areas of myocardial fibrosis. Hence, CMR represents a first etiological non-invasive step, which could be helpful to distinguish between ischemic and non-ischemic DCM.

In adult patients, various studies demonstrated not only a diagnostic but also a prognostic role of LGE especially in non-ischemic disease (5, 8–11).

Cardiac magnetic resonance assessment of myocardial fibrosis has been reported to be useful in patients with congenital heart disease (12–15) and cardiomyopathies; however, data about LGE imaging in children are still limited. Pediatric patients with DCM show a pattern of LGE distribution quite different compared to adults. Furthermore, children usually show a more heterogeneous pattern distribution of LGE compared to adults (15, 16).

Hence, our aim was to investigate the potential role of CMR and LGE in children affected by DCM.

## Patients and Methods

### Patient Selection

We retrospectively evaluated all patients affected by DCM who underwent cardiac MRI, between December 2014 and March 2016, at our institution.

Diagnosis of DCM was based on echocardiographic findings with LV end-diastolic dimension *Z* score >2 and LV EF <50%.

Patients younger than 8 years old underwent cardiac mitral regurgitation (MR) under general anesthesia.

This study was carried out in accordance with the recommendations of the “Ethical Committee of the Ospedale Pediatrico Bambino Gesù” with written informed consent from all subjects. All subjects gave written informed consent in accordance with the Declaration of Helsinki.

### CMR Acquisition Protocol

Cardiac magnetic resonance was performed with 1.5 T magnet (Aera, Siemens, Erlangen, Germany). Enrolled patients met the eligibility requirements, according to the guidelines of the American College of Radiology of 2013 (17). Patients with DCM secondary to other cardiac conditions and with glomerular filtration rate <30 ml/min/1.73 m^2^, or frequent ventricular arrhythmias, were excluded from the study.

Our protocol included cine steady-state free precession (SSFP) sequences acquired on the long-axis cardiac planes: four chambers, two chambers, and three chambers (FOV 400 × 310 mm^2^, slice thickness 6–8 mm, acquisition matrix 256 × 173, voxel size 1.5 mm × 1.5 mm × 7 mm, echo/repetition time (TE/TR) 1.1/40 ms, readout bandwidth 930 Hz/pixel, and flip angle 69°), followed by a “stack” of contiguous SSFP cine images, with the same technical parameters, acquired along cardiac short axis, to cover the whole ventricle—from base to apex.

After 10–15 min of intravenous administration of contrast agent (DOTAREM, Roissy, Guerbet, France), at 0.2 mmol/kg, T1-weighted inversion recovery sequences [FOV 400 × 290 mm^2^, slice thickness 6–8 mm, acquisition matrix 256 × 156, voxel size 1.3 × 1.3 × 7, TE/TR 3 ms/747 ms, flip angle 25°, and inversion time (TI) 250–425 ms], were acquired along the same planes of the cine SSFP images, in order to evaluate the presence of LGE.

To determine the correct TI, for nulling normal myocardial signal intensity, a look-locker sequence was acquired in one short-axis plane.

Steady-state free precession cine images and T1 inversion recovery sequence for determining LGE, both were acquired in expiratory apnea.

### Left Ventricular Systolic Function

The cine SSFP acquisitions acquired in short axis were transferred to an off-line workstation (CMR42, Circle Cardiovascular Imaging, Calgary, AB, Canada) and analyzed using the Simpson rule. End-diastolic volume (EDV), end-systolic volume (ESV), and ejection fraction (EF) were calculated for each patient. Subsequently, both EDV and ESV have been indexed for body surface area (BSA).

### MR and Left Atrial Dilatation

Mitral regurgitation was quantitatively assessed from LV stroke volume and phase contrast velocity mapping flow measurements in the aorta acquired during free-breathing (18). MR regurgitant volume was calculated as LV stroke volume (ml/beat)—aortic forward flow (mL/beat). MR regurgitant fraction (RF) was calculated as: [regurgitant volume (mL/beat) × 100]/LV stroke volume (mL/beat). MR when present was classified as follows: mild (RF < 20%), moderate (20% ≤ RF < 40%), and severe (RF ≥ 40%).

Left atrial dilatation was qualitatively assessed on cine SSFP sequences acquired, respectively, on the three different long axis view (four, two, and three chambers).

### Echocardiographic Assessment

All patients underwent a complete transthoracic echocardiographic examination using a Philips iE33 machine (Philips Medical Systems, Andover, MA, USA). Standard Doppler analysis was performed to obtain LV inflow velocities at the mitral valve tips, including peak early diastolic filling (*E*) and late diastolic peak velocities (*A*). Tissue Doppler analysis was performed to obtain longitudinal early diastolic (*e*′) myocardial velocity calculated on the septum and lateral wall. Ratio of *E/e*′ was derived to obtain an estimate of LV filling pressure. Speckle tracking imaging was used to obtain longitudinal (*L*ε) strain of the LV. Longitudinal strain were obtained from the analysis of three consecutive beats from the apical four-chamber window and expressed as percent of systolic deformation.

### Clinical Follow-up

The whole population was followed up for major adverse cardiac events (MACE) defined as a composite endpoint of death, transplant, and listing for heart transplant. The median follow-up was 17 months.

### Statistical Analysis

Analysis was performed using a commercial software (IBM SPSS Statistics for Macintosh, Version 20.0; IBM Corp., Armonk, NY, USA).

The distribution of indexed end diastolic volume (EDVi), indexed end-systolic volume (ESVi), LV EF, *E/e*′ ratio, and global longitudinal strain was established using the Shapiro–Wilk test. Subsequently Mann–Whitney *U* test was performed in order to assess a statistical significative difference between patients positive and negative for the presence of myocardial LGE. One-way ANOVA was used to compare severity MR and atrial dilatation in both groups. A cutoff value of *p* < 0.05 was considered statistically significant.

## Results

Fifteen patients were enrolled. Mean age was 8 ± 6 years. Main demographic data of the overall population are resumed in Table 1.

**Table 1:** **Main demographic data of the cohort population**.

	Patients (*n* = 15)
Età	8.4 ± 6.2 anni
Age, years (mean ± SD)	8 ± 6
Male sex, *n* (%)	6 (40)
Height, cm (mean ± SD)	119 ± 38
Weight, kg (mean ± SD)	29 ± 22
BSA (mean ± SD)	1 ± 0.5

**Figure 1:**
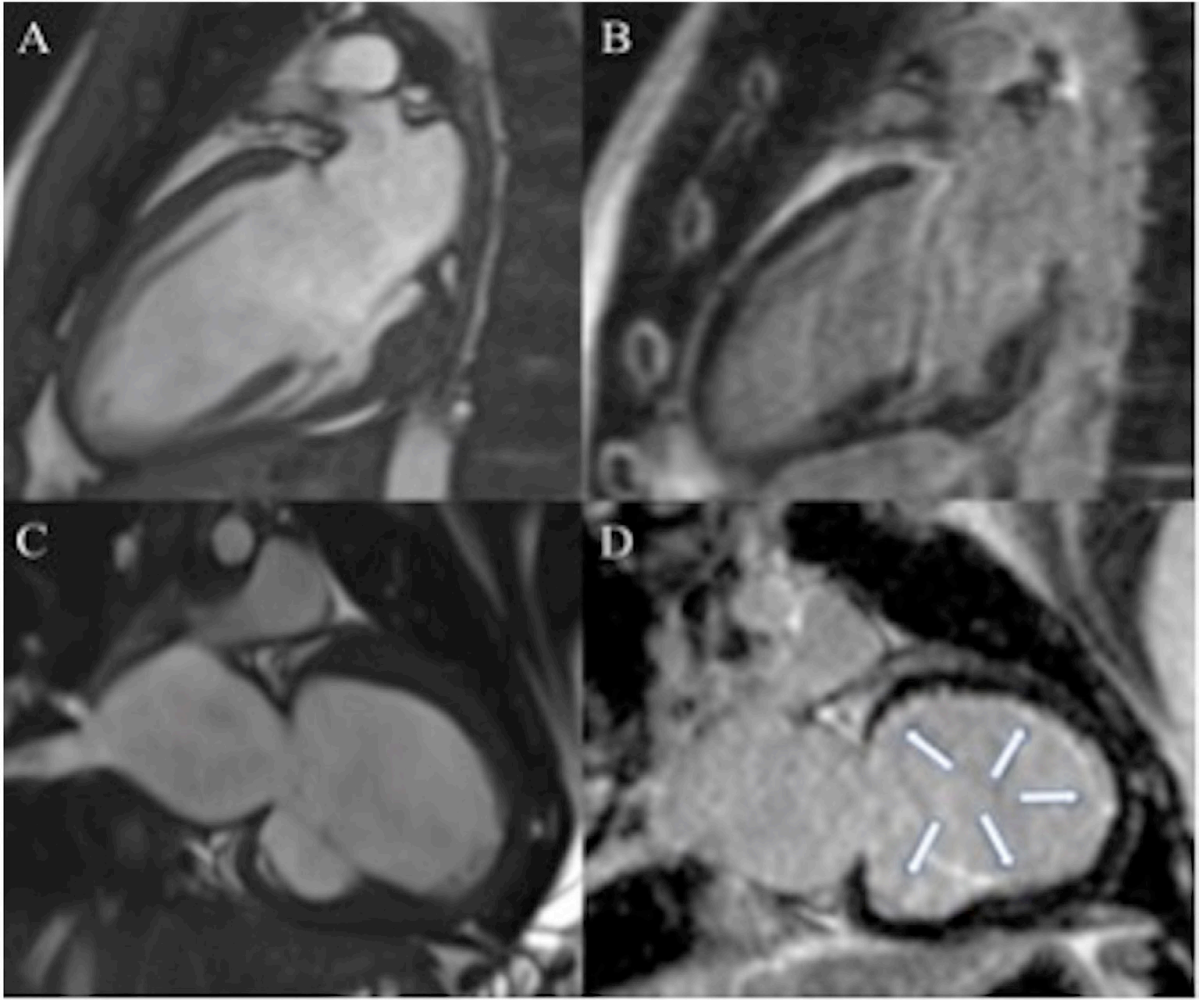
**Seventeen-year-old patient with dilated cardiomyopathy (DCM) (A,B)**. Cine steady-state free precession (SSFP), in two chambers view, shows dilated left ventricle (LV) **(A)** in absence of late gadolinium enhancement (LGE) **(B)**. Three years old patient with DCM **(C,D)**. Dilated LV in two chambers view on cine SSFP **(C)**. Diffuse subendocardial LGE is visible in two chambers view [arrows **(D)**].

**Figure 2:**
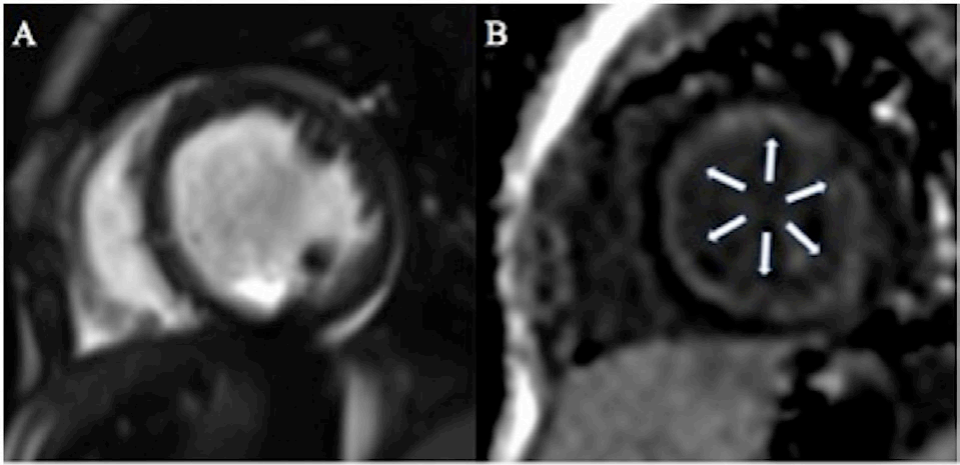
**One-year-old patient with dilated cardiomyopathy**. Cine steady-state free precession on short axis view **(A)** shows dilated left ventricle with associated diffuse global subendocardial late gadolinium enhancement [arrows **(B)**].

Within the whole population, the mean LV EF was 38 ± 11%, LV end-diastolic indexed volume was 111 ± 51 ml/m^2^. In seven patients (47%), there was significant myocardial LGE. A global diffuse (involving majority of LV segments) subendocardial pattern was evident in all patients presenting LGE (7/7, 100%) (Figures 1 and 2). Main CMR findings are summarized in Table 2.

**Table 2:** **Left ventricular volumes and function in overall population**.

	Patients (*n* = 15)
EDV, ml (mean ± SD)	106 ± 72
EDVi, ml/m^2^ (mean ± SD)	111 ± 51
ESV, ml (mean ± SD)	67 ± 48
ESVi, ml/m^2^ (mean ± SD)	71 ± 40
EF, % (mean ± SD)	38 ± 11
LA dilation, *n* (%)	5 (33)
Mitral regurgitation, *n* (%)	
Absent	6 (40)
Mild	5 (34)
Moderate	2 (13)
Severe	2 (13)
*E/e*′ ratio	10 ± 3
Global longitudinal strain, %	−14 ± 4
Major adverse cardiac events (*n*, %)	4 (26)
LGE (*n*, %)	7 (47)

*EDV, end-diastolic volume; EDVi, BSA-indexed end-diastolic volume; ESV, end-systolic volume; ESVi, BSA-indexed end-systolic volume; EF, ejection fraction*.

The study population was divided into two groups: patients with no evidence of significant LGE (Group A, eight patients) and patients with the presence of diffuse subendocardial LGE (Group B, seven patients). The two subgroups were well matched for age and sex.

In Group A, the main findings were (values expressed as average ± standard deviation): age 7 ± 6 years, indexed EDV 96 ± 33 ml, indexed ESV 56 ± 29 ml, LV EF 45 ± 10%, global longitudinal strain = −16 ± 5%, and *E/e*′ ratio = 10 ± 3. While Group B showed the following data: age 9 ± 4 years, indexed EDV 130 ± 60 ml, indexed ESV 89 ± 43 ml, LV EF 31 ± 6%, global longitudinal strain = −13 ± 4%, and *E/e*′ ratio = 9 ± 3.

Main demographic data, CMR, and echocardiography findings among the two subgroups are shown in Table 3.

**Table 3:** **Comparison of left ventricular volumes and function between Group A and Group B**.

	Group A (*n* = 8)	Group B (*n* = 7)	*p* Value
Male sex, *n* (%)	3 (38%)	3 (43%)	0.5
Age, years (mean ± SD)	7 ± 6	9 ± 4	0.8
EDVi, ml/m^2^ (mean ± SD)	96 ± 33	130 ± 60	0.2
ESVi (ml/m^2^; mean ± SD)	56 ± 29	89 ± 43	0.2
EF (*n*, %)	45 ± 10	31 ± 6	0.03
LA dilation (*n*, %)	2 (25)	3 (42)	0.5
MR at least moderate (*n*, %)	0 (0)	4 (60)	0.009
*E/e*′ ratio	10 ± 3	9 ± 3	0,9
Global longitudinal strain (%)	−16 ± 5	−13 ± 4	0.03

*EDVi, end-diastolic volume; ESVi, BSA-indexed end-systolic volume; EF, left ventricle ejection fraction; LA, left atrium; MR, mitral regurgitation*.

No significative difference was observed in terms of indexed EDV (*p* = 0.2) and indexed ESV (*p* = 0.2). However, LV EF was significantly lower in Group B patients compared to Group A (*p* = 0.03). Moreover, the presence of MR—at least moderate—was significantly lower in the subgroup of patients with no significant LGE (*p* = 0.009). A significative difference of global longitudinal strain was observed between Groups A and B (*p* = 0.03).

No significative difference in terms of LA dilation (*p* = 0.5), possible indirect sign of diastolic dysfunction, and *E/e*′ (*p* = 0.9) were observed between Groups A and B.

Major adverse cardiac events were more frequent on Group B [3 (38%) vs 1 (14%)], but the small sample size did not allow any survival analysis.

## Discussion

This study demonstrates that in children affected by DCM, despite comparable LV volumes, the presence of diffuse subendocardial LGE is associated with a decreased LV global systolic function and worst global longitudinal strain. Interestingly, a global diffuse subendocardial pattern, involving majority of LV segments, papillary muscles, and trabeculae, was evident in all patients presenting LGE.

A typical pediatric cardiomyopathy, quite similar to our finding, had been already described in autopsy since the 1950s, and it was called primary endocardial fibroelastosis (EFE) (19, 20). Such entity was presumed to lead to a DCM (21). Thus, although primary EFE had been previously labeled as a separate form of cardiomyopathy, in 2006 the new classification of the American Heart Association does not consider it anymore as an isolate disease and includes it in the spectrum of DCM (22). Primary EFE in the original description is characterized by the presence of ventricular dilatation, diffuse fibrous endocardial thickening, upward displacement of the papillary muscles, and valve leaflets thickening (20, 23, 24). In terms of “outcome,” EFE is characterized by a very poor prognosis in pediatric patients (25, 26).

Our findings seem to confirm this report and corroborate the hypothesis that in the presence of EFE the ventricular mechanic is compromised, leading to impaired contraction and expansibility of the cardiac cavity.

Moreover, development of this peculiar LGE pattern does not seem to be related to disease duration, since age of the two subgroups is not significantly different.

Our study also shows that significant MR is more frequent in patients with significant LGE. This finding should not be linked to a functional mechanism, since ventricular dilation is comparable in the two subgroups. Therefore, the increasing leakage seems to be related to a fibrotic involvement of the valve and subvalvar apparatus. This is in keeping with the original description of primary EFE where papillary muscles involvement and valve leaflets thickening were described. Our data hence confirm that the presence of global diffuse EFE in the setting of children affected by DCM identifies a subgroup of patients with a worse disease clinical expression.

The etiology of EFE is still debated in literature. Some articles attribute the development of this condition to an increased response of fetal myocardium in case of viral illnesses including mumps, during pregnancy (27). The other main hypothesis is that a sort of ischemic/vascular problem is the origin of this peculiar finding. This is based on the global hypoperfusion detectable as myocytolysis at the examination of autoptic specimen in these patients. However, this abnormal finding can be commonly found also in patients with DCM (21).

Curiously, between the two subgroups we could not find any difference in terms of LA dilation and *E/e*′ ratio. EFE is commonly believed to be a cause of diastolic dysfunction (28, 29). Our finding could be due to the poor sample size. Nevertheless, as it is well known for adult population, diastolic dysfunction might occur later in the natural history of children with DCM.

We must admit that the main limitation of this study is the small number of our cohort, which does not allow any definitive conclusion. Nevertheless, it should be emphasized that indication to CMR for a children affected by DCM is not so straightforward. Since on average general anesthesia is required for patients younger than 8 years old, balancing possible risks and benefits, the number of patients undergoing CMR in this setting is little worldwide, especially in the infancy.

Furthermore, we should admit that the evaluation of MR calculated with free breathing phase contrast may represent a bias if compared with stroke volume deducted by breath-hold cine images. In effect, it has been demonstrated that a trend to have higher stroke volume during free breathing phase contrast sequences if compared to the breath-hold ones (30). This could influence the RF of mitral valve that we calculated with magnetic resonance, although a concordance between data acquired on echocardiography and magnetic resonance in our cohort was observed.

From a clinical point of view, a trend of increased likelihood for developing MACE in population with positive LGE was found. However, the small sample size did not allow any specific survival analysis.

The prognostic role of LGE in children affected by DCM in effect is likely one of the most appealing future perspective, which could arise from our pilot study. Clearly, our initial findings should be confirmed on a wider analysis, performed on a larger cohort of patients.

## Conclusion

In our population of children affected by DCM, LGE is frequent (47%), and it shows a characteristic global diffuse subendocardial pattern. Despite comparable LV volumes, the presence of fibrosis seems to play a key role in these patients, determining a worst clinical expression of disease. If LGE is present, patients have a significant lower global systolic function and more frequently show a significant MR. More data confirming these findings on a larger population and investigating the prognostic role of LGE in this setting are needed.

## Ethics Statement

The authors declare that this study was performed in accordance with the research ethical guidelines. The study was conducted in retrospective analysis.

## Author Contributions

GM, PC, and AS wrote the manuscript. DM, CN, and TS collected data and analyzed the images. GR, MC, and BL analyzed data and were involved in manuscript editing.

## Conflict of Interest Statement

The authors declare that the research was conducted in the absence of any commercial or financial relationships that could be construed as a potential conflict of interest.
